# Reframing Hyaluronidase in Aesthetic Medicine: From “Dissolving Filler” to “Modifying Filler”

**DOI:** 10.1093/asj/sjag022

**Published:** 2026-01-27

**Authors:** Steven Harris, Steven Weiner

Hyaluronidase is one of the most widely used agents in aesthetic medicine. It is indispensable for managing vascular occlusion and is increasingly used electively to address overfilling, contour abnormalities, and filler spread (commonly referred to as filler “migration”). Recent guidelines have refined dosing strategies for both elective and emergency scenarios.^1-3^

Despite this central role, the prevailing terminology of “dissolving filler” is scientifically imprecise and clinically misleading. It assumes complete, uniform, and predictable elimination of hyaluronic acid (HA) filler—an assumption not supported by filler rheology, HA biology, ultrasound findings, or magnetic resonance imaging (MRI).^4-11^ Clinicians frequently observe that aesthetic improvement exceeds what imaging demonstrates, whereas residual HA persists even after multiple treatment sessions. These discrepancies indicate that hyaluronidase interacts with a filler–tissue complex rather than isolated, discrete deposits of gel.

This article is intended as a narrative editorial, synthesizing biological principles, imaging observations, and clinical experience, rather than a hypothesis-driven experimental or controlled clinical study. It argues for abandoning the outdated language of “dissolving” in favor of a biologically accurate and clinically responsible conceptualization: “filler modification.” Clinically meaningful aesthetic improvement following hyaluronidase often coexists with persistent HA visible on ultrasound or MRI.^7-9,11^ At a molecular level, hyaluronidase cleaves HA polymers into shorter fragments, reducing viscosity, cohesivity, and hydrophilicity. Cross-linking slows, but does not prevent, enzymatic degradation. Rheological studies demonstrate progressive softening and loss of gel integrity rather than complete erasure.^4^ Comparative in vitro studies of commercial HA fillers show substantial variability in susceptibility to hyaluronidase depending on manufacturing processes and gel structure.^5^ These findings challenge the assumption of predictable or complete dissolution.

Endogenous HA biology further contradicts the common “dissolve and replace” narrative. Native dermal HA has a half-life of ∼24 h and undergoes continuous metabolic turnover through multiple enzymatic pathways, including HYAL1, HYAL2, TMEM2, and CEMIP as well as nonenzymatic mechanisms such as oxidative degradation.^6,10^ Importantly, this baseline turnover is not upregulated to compensate for acute loss of native HA following hyaluronidase exposure, nor does it restore tissue architecture or volume to pretreatment states. Consequently, the notion of “dissolve and replace” is physiologically inaccurate.

In cases of chronic overfilling, clinicians encounter a composite matrix consisting of partially degraded filler, changes consistent with fibrosis and septal thickening, chronic edema driven by prolonged hydrophilicity, and mechanically redistributed gel. Hyaluronidase interacts with and modifies this complex by reducing swelling, loosening gel structure, improving tissue pliability, and unmasking underlying anatomy. Clinical improvement therefore reflects modification of the filler–tissue complex rather than eradication of every HA molecule.

Repeated or long-standing filler injections do not persist as discrete, localized deposits. Instead, filler becomes integrated into a dynamic tissue environment shaped by hydration behavior, fibroplasia, mechanical forces such as facial movement and compression, and compartmental spread or migration along anatomical planes.^7,8,11-13^

This model explains why HA in some regions responds readily to hyaluronidase while others are resistant, why multiple treatment sessions are often required, and why imaging frequently demonstrates residual filler despite clinical improvement. Promising “complete dissolution” is therefore scientifically untenable, particularly in the context of chronic, integrated filler–tissue systems (Figures 1, 2).

**Figure 1.**
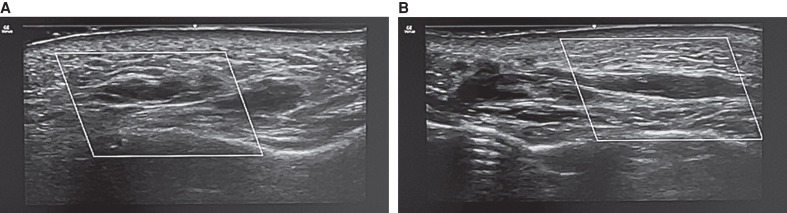
Ultrasound demonstrating hyaluronic acid filler tracking along the superficial muscloaponeurotic system plane from the nasolabial region (A) toward the infraorbital area (B). This illustrates plane-based migration along paths of least resistance.

**Figure 2.**
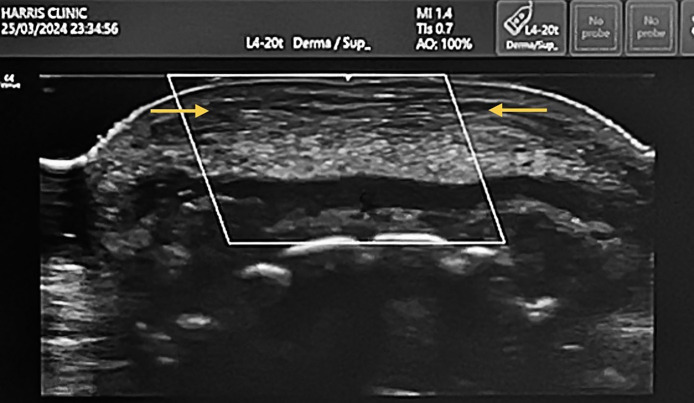
High-frequency ultrasound of the lower lip showing unintended intramuscular (pars marginalis) hyaluronic acid filler deposition (between arrows), consistent with chronic overfilling patterns.

Ultrasound provides valuable insight into this complexity, high-frequency imaging consistently demonstrates heterogeneous filler distribution, frequent intramuscular placement even when unintended, compartmental spread along fascial or muscular planes, and partial rather than complete loss of echogenic material following hyaluronidase.^7,8,11,14,15^ These findings support variability rather than a binary “dissolved vs not dissolved” outcome.

However, ultrasound findings are descriptive rather than definitive, and echogenic patterns are not specific—hypoechoic areas may represent residual HA, edema, or fluid, whereas fibrosis is typically hyperechoic and may partially obscure underlying filler. Chronic filler often produces mixed echogenicity that requires contextual interpretation, and both acquisition and interpretation are highly operator dependent. Equipment quality also varies substantially; high-frequency medical ultrasound systems (15-22 MHz) offer superior axial and lateral resolution compared with lower-resolution handheld devices, which may struggle to differentiate filler from edema or adjacent hypoechoic structures such as deep fat, or muscle, and may poorly visualize subtle fibrotic change. At present, there are no consensus ultrasound criteria for “complete resolution,” as residual gel, edema, and fibrosis can coexist even when clinical appearance improves (Figure 3 and Videos 1 and 2).

**Figure 3.**
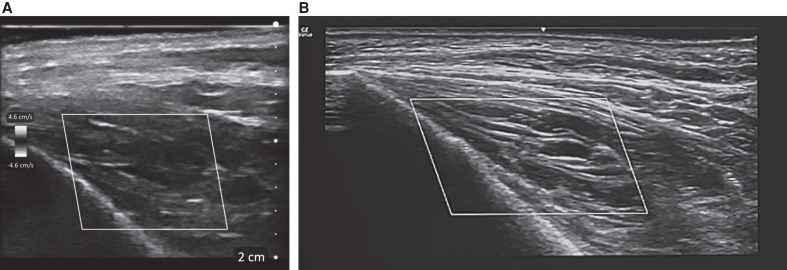
Comparison of a handheld ultrasound device. (A) Clarius L20 (Clarius Mobile Health, Vancouver, BC, Canada) and a high-resolution medical ultrasound system. (B) GE Venue Go, L4-20 probe (GE Healthcare, Chicago, IL), demonstrating differences in axial and lateral resolution.

MRI studies, including a 33-patient series demonstrating long-term persistence of HA, confirm that filler may remain visible many years after injection.^8,9,11^ However, MRI has important limitations. There is no standardized HA-specific imaging protocol, and T_2_-hyperintense signal overlaps with edema, inflammation, and fluid collections. Radiological interpretation therefore varies widely, and MRI cannot quantify how much filler remains.^9^ Imaging thus supports 1 robust conclusion: filler persistence is more complex than previously assumed, and imaging findings should not be interpreted as quantitative or definitive markers of clinical treatment success (Figure 4).

**Figure 4.**
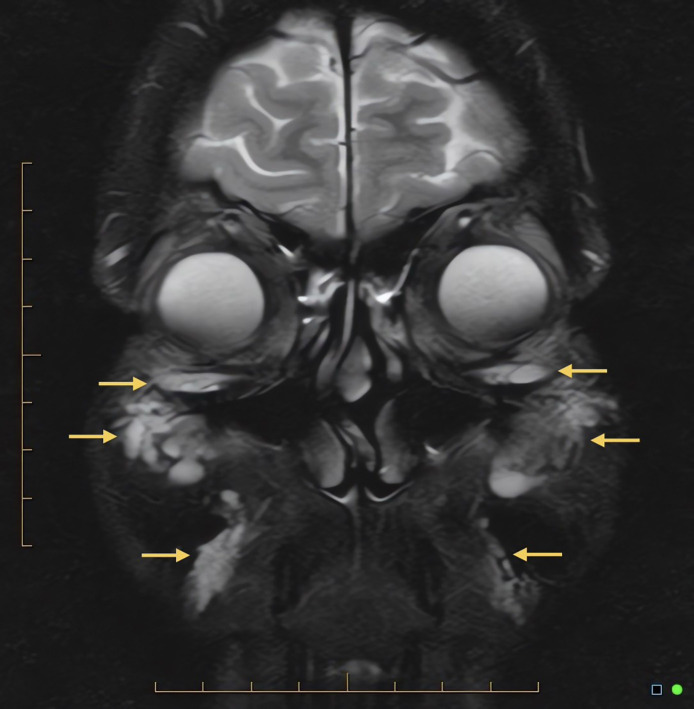
Coronal T_2_-weighted MRI demonstrating multiple T_2_-hyperintense foci (arrows) in the infraorbital, prezygomatic, deep cheek, and marionette regions. These signals are consistent with residual hyaluronic acid filler but may overlap with edema or other soft-tissue fluid.

The language of “dissolving” misrepresents enzymatic behavior, promotes unrealistic patient expectations, and increases medico-legal vulnerability when imaging later demonstrates persistence. Hyaluronidase cleaves HA but does not destroy collagen, fat, fascia, ligaments, or other structural tissues.^15^ Years of accumulated filler embedded within altered tissue architecture are incompatible with a simplistic dissolve model (Figure 5).

**Figure 5.**
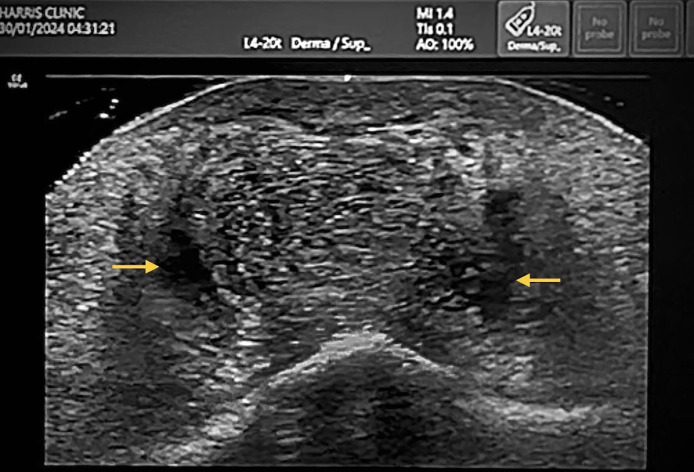
Ultrasound of the chin demonstrating chronic filler-related changes, including persistent hyaluronic acid material (arrows), interstitial edema, and hyperechoic fibrotic septa.

Psychological factors further complicate interpretation. Although most patients view hyaluronidase positively, a minority, often active in online forums, misattribute normal anatomical variation, age-related change, or the secondary effects of chronic filler (such as tissue expansion or stretch) to “hyaluronidase damage.” Qualitative research on perceptual drift demonstrates that patients may normalize distorted proportions after years of filler, such that restoration of baseline anatomy can be perceived as loss or harm.^13^ These observations are interpretative and highlight the need for prospective research, but they underscore the importance of precise, biologically grounded language when counseling patients.

High-dose hyaluronidase remains the established standard of care for vascular occlusion.^1,3^ Elective treatment, however, differs fundamentally in context. Chronic filler behaves differently from recent injections, outcomes depend on the surrounding tissue environment, and aesthetic restoration often requires staged intervention. Reframing elective treatment as filler modification rather than dissolution more accurately reflects these realities and influences consultation management, informed consent, dosing strategies, treatment timing, and outcome assessment.

At present, no validated aesthetic scales, volumetric measurements, or standardized patient-reported outcome measures exist to quantify the structural changes described following hyaluronidase treatment. Similarly, the proposed terminological shift from “dissolving” to “modifying” filler has not been empirically tested. It is offered as a conceptual framework grounded in biological behavior, imaging variability, and clinical experience rather than as a data-driven outcome comparison. Practice patterns, regulatory environments, and imaging expertise also vary internationally, and the concepts presented should be interpreted within local clinical contexts.

Hyaluronidase does not “dissolve” HA filler in the simplistic sense implied by current terminology. Instead, it modifies a complex filler–tissue system by altering gel structure, hydration, mechanical properties, and anatomical visibility. Adopting the language of modification aligns clinical practice with biological reality, improves patient understanding, reduces medico-legal risk, and supports more transparent, responsible aesthetic care.

## Supplementary Material

sjag022_Supplementary_Data
